# Spatial–temporal analysis of pulmonary tuberculosis among students in the Zhejiang Province of China from 2007–2020

**DOI:** 10.3389/fpubh.2023.1114248

**Published:** 2023-02-09

**Authors:** Mengdie Zhang, Songhua Chen, Dan Luo, Bin Chen, Yu Zhang, Wei Wang, Qian Wu, Kui Liu, Hongmei Wang, Jianmin Jiang

**Affiliations:** ^1^Department of Social Medicine of School of Public Health and Department of Pharmacy of the First Affiliated Hospital, Zhejiang University School of Medicine, Hangzhou, Zhejiang, China; ^2^Department of Tuberculosis Control and Prevention, Zhejiang Provincial Center for Disease Control and Prevention, Hangzhou, Zhejiang, China; ^3^Department of Public Health, Hangzhou Medical College, Hangzhou, Zhejiang, China; ^4^Key Laboratory of Vaccine, Prevention and Control of Infectious Disease of Zhejiang Province, Zhejiang Provincial Center for Disease Control and Prevention, Hangzhou, Zhejiang, China

**Keywords:** pulmonary tuberculosis, spatial–temporal, Joinpoint regression, student, epidemiology

## Abstract

**Background:**

Pulmonary tuberculosis (PTB) is a serious chronic communicable disease that causes a significant disease burden in China; however, few studies have described its spatial epidemiological features in students.

**Methods:**

Data of all notified PTB cases from 2007 to 2020 in the student population were collected in the Zhejiang Province, China using the available TB Management Information System. Analyses including time trend, spatial autocorrelation, and spatial–temporal analysis were performed to identify temporal trends, hotspots, and clustering, respectively.

**Results:**

A total of 17,500 PTB cases were identified among students in the Zhejiang Province during the study period, accounting for 3.75% of all notified PTB cases. The health-seeking delay rate was 45.32%. There was a decreasing trend in PTB notifications throughout the period; clustering of cases was seen in the western area of Zhejiang Province. Additionally, one most likely cluster along with three secondary clusters were identified by spatial–temporal analysis.

**Conclusion:**

Although was a downward trend in PTB notifications among students during the time period, an upward trend was seen in bacteriologically confirmed cases since 2017. The risk of PTB was higher among senior high school and above than of junior high school. The western area of Zhejiang Province was the highest PTB risk settings for students, and more comprehensive interventions should be strengthened such as admission screening and routine health monitoring to improve early identification of PTB.

## 1. Introduction

Due to the serious health risk that pulmonary tuberculosis (PTB) poses, PTB has aroused substantial public concern worldwide. The current case detection ratio globally is insufficient to attain the goal of ending TB by 2035 (1–4). According to the Global Tuberculosis Report released in 2021 (5), approximately 9.9 million people were diagnosed with TB in 2020, with an incidence of 127 cases per 100,000 population globally. China, as one of the 30 high-burden countries, has 842,000 newly diagnosed PTB cases annually, with an incidence rate of 59 cases per 100,000 persons (5). Now, more interventions and implementations have been recommended for target populations, including the older adults and people with diabetes mellitus, as a priority to reduce the prevalence. However, compared with these target groups, students have also shown a considerable risk of PTB control (6). Previous studies reported a total of 44,721 students with PTB in China in 2020, with a notified incidence rate of 15.85 cases per 100,000 (7). Although PTB incidence was significantly lower in students than the general population, the onset of PTB among this group may attract more attention arousing public health incidents. The high density of students in schools and crowded living environments may facilitate the transmission of PTB among students, even leading to a serious outbreak in the short term (8–11).

Zhejiang Province is a developed region located in the eastern area of China. The notified incidence of PTB showed a downward trend from 75.38 cases per 100,000 in 2009 to 52.25 cases per 100,000 in 2018 (12). There were approximately 27,000 people newly ill with PTB per year (13). Meanwhile, spatial–temporal analysis was widely used to characterize the epidemiological distribution and aggregation of infectious diseases in space and time, although few studies have explored the spatial epidemiological characteristics of PTB among students (14).

In this study, we aimed to explore the epidemiological characteristics of PTB among students in the Zhejiang Province. We further attempted to identify PTB clustering among this special population, which may provide valuable clues to promote the further control and intervention of PTB epidemic in students.

## 2. Methods

### 2.1. Overview of the study area

Zhejiang Province is in the eastern region of China with a permanent population of 64.57 million in 2020, which covers an area of 100,000 square km, including plains, mountains, seashores, islands, and lakes (15). There are two sub-provincial cities of Hangzhou and Ningbo, and nine prefecture-level cities, including Wenzhou, Huzhou, Shaoxing, Jiaxing, Taizhou, Quzhou, Jinhua, Lishui, and Zhoushan (16). There are a total of 90 counties/cities within the Zhejiang Province.

### 2.2. Data source

Data for all notified PTB cases among students in Zhejiang Province were collected in the Tuberculosis Information Management System (TBIMS) from January 2007 to December 2020. We excluded extrapulmonary TB cases and cases identified as nontuberculous mycobacteria (17). All students included kindergartens, primary schools, high schools, and universities (18). Details for each PTB case included basic demographic information, clinical diagnosis information, laboratory outcomes, and treatment outcomes. Annual student population data for each administrative district were obtained from the Zhejiang Statistical Yearbook, education administrative department and Local Statistical Yearbook.

### 2.3. Definitions

Combined with the features of age group and grade in China, students aged 3–6, 7–12, 13–15, and 16–18 years old were defined as kindergarten, primary school, and junior high, and senior high school students. Students greater than 19 years old were denoted as college students and above in this study. Notified PTB cases included laboratory-confirmed and clinically diagnosed cases. Clinically diagnosed PTB cases were defined as those with PTB-specialized chest imaging, clinical manifestations (coughing, expectoration for ≥2 weeks, or hemoptysis), and no response to diagnostic anti-inflammatory therapy with negative laboratory test results or missing results (anti-TB drug was excluded) (19). Laboratory confirmation of PTB was based on sputum smear, culture, and GeneXpert results indicating infection with M. tuberculosis. Completion of treatment course was defined as: (1) clinically diagnosed PTB patients completed the standardized course and had negative sputum smear/culture results and (2) laboratory-confirmed patients who completed the prescribed course of treatment, with a negative sputum test result at the latest, and no sputum test result at the end of the course of treatment. Cure was defined as persistent negative sputum smear or culture results for two times, one of which was at the completion of the standardized course of treatment in laboratory-confirmed patients (20). Multidrug-resistant tuberculosis (MDR-TB) was defined as tuberculosis that was resistant to two or more drugs, such as isoniazid and rifampin (21). Health-seeking delay was defined as the time interval from the occurrence of the first symptoms to the first visit to a designated hospital, when the first visit was >2 weeks after the occurrence of the first symptoms. Diagnostic delay referred to the interval between the visit to a designated hospital and diagnosis of PTB, if the diagnosis took >2 weeks (22).

### 2.4. General characteristics of PTB in students

Based on bacteriological or clinical diagnoses, the results of notified PTB cases among students were described in terms of sex, age group, city, anti-TB treatment, source of patients, classification of treatment, delays in seeking medical care, and treatment outcome.

### 2.5. Time trend analysis

We used the Joinpoint regression method to assess the yearly time trend of the notification rates of PTB and evaluate the annual percentage changes (APCs) and their confidence intervals (23). The Joinpoint regression model builds a segmented regression based on the temporal characteristics of the disease distribution. By partitioning the study time into different intervals, the Joinpoint regression method can fit and optimize the trend for each interval, ultimately evaluating the details among different interval-specific disease characteristics (24). The model analyzes statistically significant turning points and calculates the corresponding *t*-value and *P*-value through Monte Carlo permutation testing and selects the best-fit models according to Bayesian information criterion. In this study, we took the nature logarithm of annual notification incidence of PTB among students as the dependent variable and the year of notification as the independent variable. The trend change in this time segment was statistically significant when the APC had a *P* < 0.05 and the 95% confidence interval (CI) did not include 0 (25).

### 2.6. Spatial autocorrelation analysis

Spatial autocorrelation analysis, which includes global spatial autocorrelation and local spatial autocorrelation, is used to reveal the spatial distribution patterns of diseases, and identify disease risk areas. The global spatial autocorrelation analysis is a description of the spatial characteristics of the attribute values of the entire region and determines the aggregation characteristics of the studied variables in the overall space (26). Moran's I was used to assess the spatial autocorrelation, taking values in the range of−1 to 1. Moran's I >0 indicates a positive spatial autocorrelation, <0 means a negative autocorrelation, and 0 indicates random spatial distribution (no spatial autocorrelation). Moran's I of larger absolute values indicates a stronger spatial autocorrelation (27). A Moran's I was statistically significant at *P* < 0.05 and Z-score ≥ 1.96. Meanwhile, local spatial autocorrelation analysis was used to identify the spatial correlation between the study variables and the area variables of the neighboring regions (28). Local indicator of spatial autocorrelation (LISA) was used to test the local autocorrelation results to evaluate the existence of local clusters and detect regions with significant spatial correlations (29).

### 2.7. Spatial–temporal scan statistic

Kulldorff's space–time scan statistics are based on the Poisson probability distribution model to detect the spatial–temporal aggregation of diseases. The space–time scan statistic uses the moving windows to create a cylinder with the bottom surface as the scan area, height as the possible clustering time, and radius as the scan risk population (30). The size and location of scan window was constantly changed in space and time for searching possible clusters. In this method, the expected incidence is calculated based on the actual incidence number and population size of each window, and it is compared with the actual incidence number to build the log likelihood ratio (LLR) for identifying clusters using the Monte Carlo randomization method to examine the statistically significant cluster at 95% CI (31).

### 2.8. Statistic analysis

Descriptive analysis was conducted using R software (version 4.2.1, R Foundation for Statistical Computing) and Microsoft Excel. Joinpoint software (Joinpoint Regression Program, Version 4.9.1.0, April 2022; Statistical Methodology and Applications Branch, Surveillance Research Program, National Cancer Institute) was used to calculate the APC and its 95% CI. Moran's I was calculated using GeoDa (version 1.20) and SaTScan software (version 10.1, Boston, MA, USA) was used to determine the spatial and temporal aggregation of PTB. All visual results were presented using ArcGIS software (version 10.8, SERI Inc., Redlands, CA, USA).

### 2.9. Ethics statement

Our study was approved by the ethics committee of the Zhejiang Center for Disease Control and Prevention and exempted from the requirement of informed consent. All personal information in this study was kept confidential as required.

## 3. Results

### 3.1. General characteristics of PTB in students

**Figure 1:**
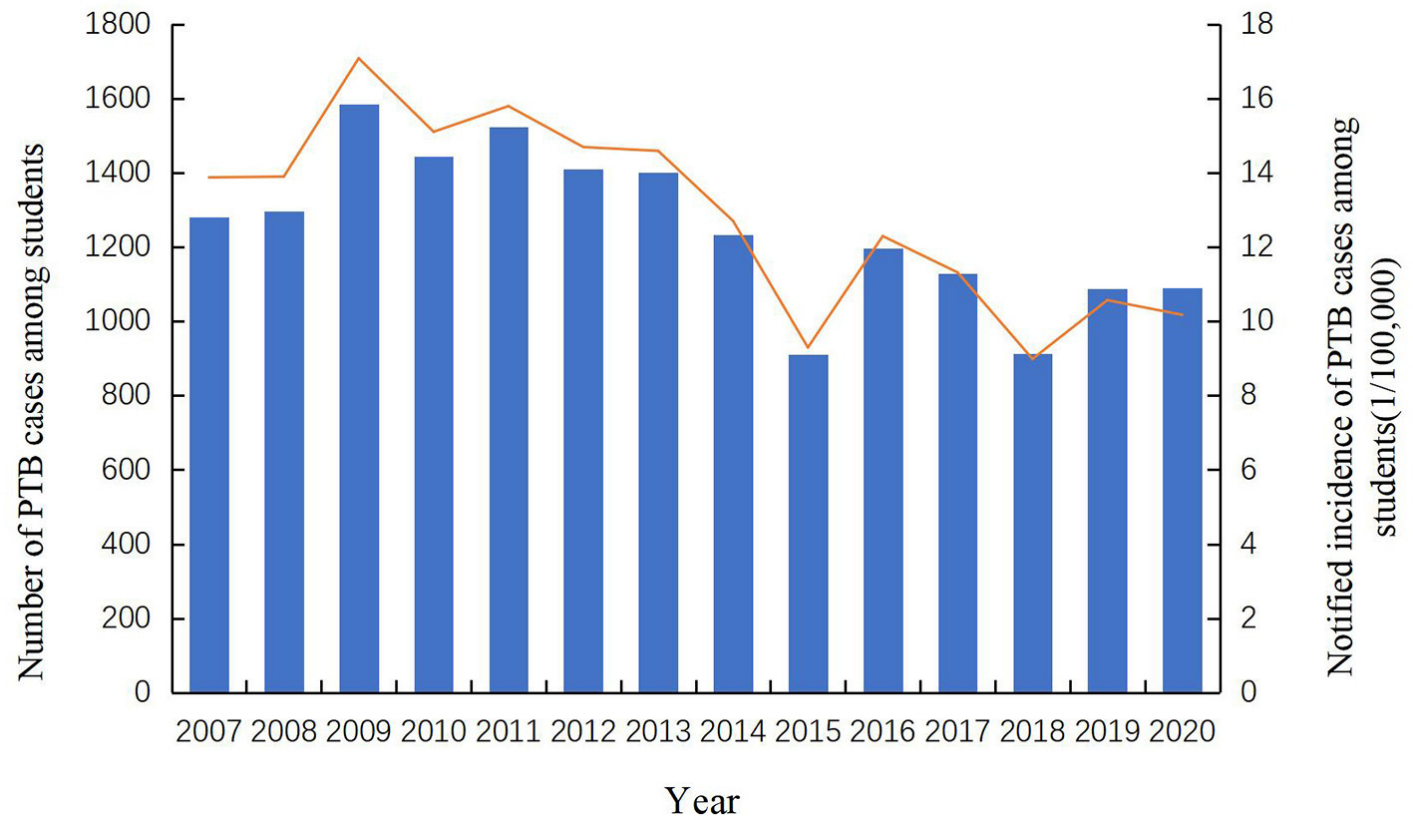
Trend of notified PTB incidence and notified number of PTB cases by years.

**Figure 2:**
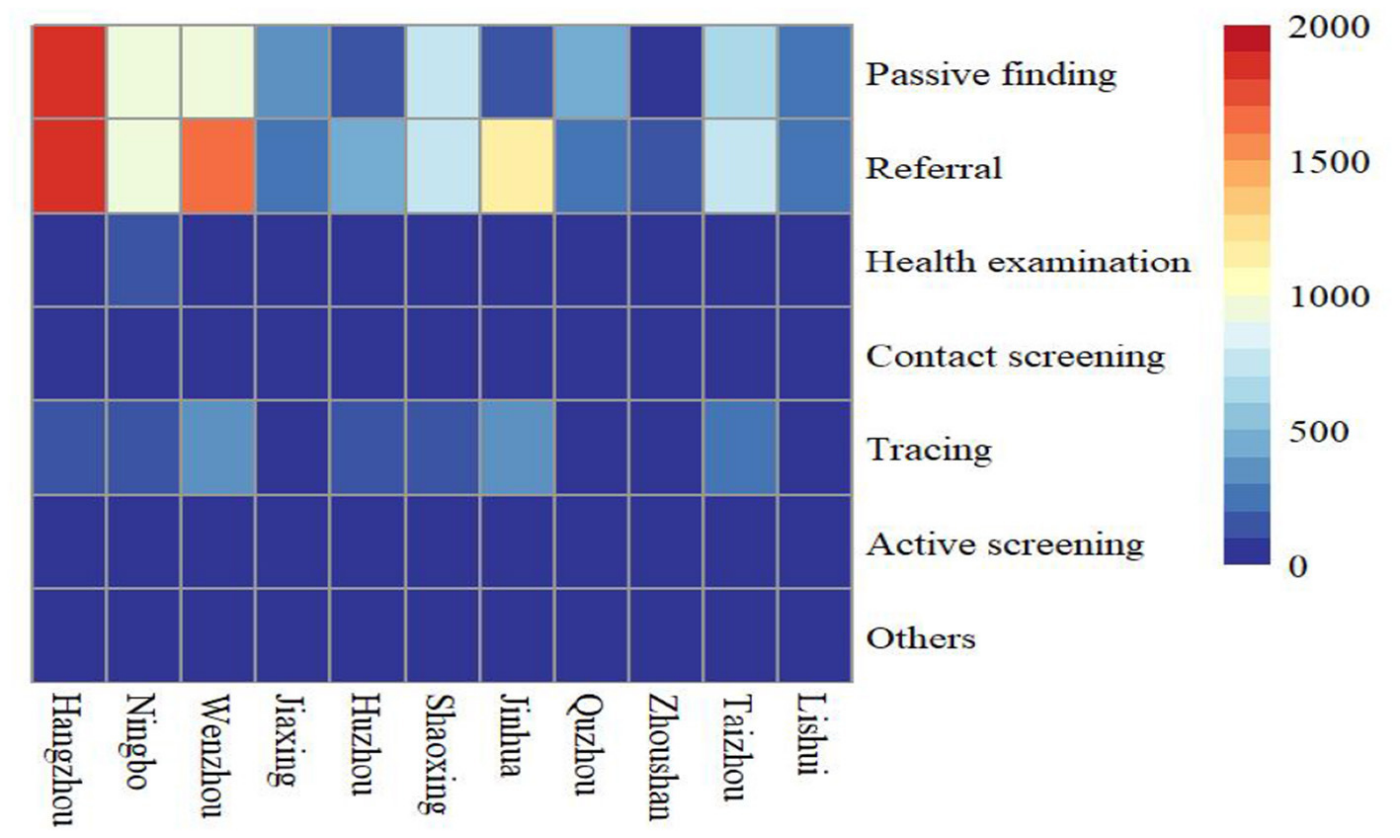
Heatmap of patient sources in different cities from 2007 to 2020 (Different colors indicate different notified number of student PTB).

A total of 17,500 PTB cases among students were notified in the Zhejiang Province from 2007 to 2020, accounting for 3.75% of all identified PTB cases (466,958). Among all diagnosed PTB cases, 68.77% (12,035) were derived from the resident population and the rest (5,465, 31.23%) were from the migrant population. The notified PTB incidence among students ranged from 9 cases per 100,000 to 17 cases per 100,000, with an average annual notified incidence of 12.89 cases per 100,000 population (Figure 1). From 2007 to 2013, the annual incidence of student PTB remained higher than the average incidence during the study period. A peak notified PTB incidence of 17.08 per 100,000 was observed in 2009. Among all the diagnosed PTB cases, 59.69% (10,445) were male and 40.31% (7,055) were female, with a male to female ratio of 1.48:1. In terms of age distribution, 12.86% were 13–15 years old, 39.90% were 16–18 years old and 43.65% were more than 19 years old. In terms of geographic distribution, more than 10% of all notified student cases were found in Hangzhou, Wenzhou, Ningbo, and Jinhua; the highest proportion of PTB bacteriological diagnosis (272, 40.42%) was observed in Jiaxing city while the highest proportion of clinical diagnosis (605, 76.01%) was observed in Quzhou city. Referral and passive findings were the main sources of student PTB in the Zhejiang province (Figure 2). 97.69% of PTB cases in students were initially treated and 70.16% were clinically diagnosed. Almost all student PTB patients received anti-TB treatment and nearly 95% of PTB cases completed PTB treatment or were cured. While 7,931 student cases (45.32%) experienced health-seeking delays, there was a low rate of diagnostic delay (2,453, 14.02%). In addition, 24.49% (4,286) of the students' cases had a treatment duration of ≥9 months (Table 1).

**Table 1:** Epidemiological characteristics of PTB cases among students in Zhejiang Province from 2007 to 2020 (*N* = 17,500).

**Characteristic**	**Total PTB**	**Laboratory-confirmed PTB**	**Clinically diagnosed PTB**
**Sex**, ***n*** **(%)**			
Male	10,445 (59.69)	2,885 (27.62)	7,560 (72.38)
Female	7,055 (40.31)	2,371 (33.61)	4,684 (66.39)
**Grade/Age (years)**, ***n*** **(%)**			
Kindergarten students (3–6)	14 (0.08)	1 (7.14)	13 (92.86)
Primary school students (7–12)	614 (3.51)	164 (26.71)	450 (73.29)
Junior high school students (13–15)	2,251 (12.86)	893 (39.67)	1,358 (60.33)
Senior high school students (16–18)	6,983 (39.90)	2,056 (29.44)	4,927 (70.56)
College school students and above (≥19)	7,638 (43.65)	2,142 (28.04)	5,496 (71.96)
**City**, ***n*** **(%)**			
Hangzhou	4,005 (22.89)	967 (24.14)	3,038 (75.86)
Huzhou	784 (4.48)	269 (34.31)	515 (65.69)
Jiaxing	673 (3.85)	272 (40.42)	401 (59.58)
Jinhua	1,765 (10.09)	694 (39.32)	1,071 (60.68)
Lishui	708 (4.05)	207 (29.24)	501 (70.76)
Ningbo	2,261 (12.92)	720 (31.84)	1,541 (68.16)
Quzhou	796 (4.55)	191 (23.99)	605 (76.01)
Shaoxing	1,623 (9.27)	517 (31.85)	1,106 (68.15)
Taizhou	1,655 (9.46)	467 (28.22)	1,188 (71.78)
Wenzhou	3,030 (17.31)	895 (29.54)	2,135 (70.46)
Zhoushan	200 (1.14)	57 (28.50)	143 (71.50)
**Patient discovery method**, ***n*** **(%)**			
Health examination	643 (3.67)	105 (16.33)	538 (83.67)
Contact screening	218 (1.25)	20 (9.17)	198 (90.83)
Passive finding	6,482 (37.04)	1,875 (28.93)	4,607 (71.07)
Referral	8,424 (48.14)	2,668 (31.67)	5,756 (68.33)
Tracing	1,567 (8.95)	551 (35.16)	1,016 (64.84)
Active screening	51 (0.29)	9 (17.65)	42 (82.35)
Others	115 (0.66)	28 (24.35)	87 (75.65)
**Treatment classification**, ***n*** **(%)**			
First treatment	17,096 (97.69)	5,101 (29.84)	11,995 (70.16)
Retreatment	404 (2.31)	155 (38.37)	249 (61.63)
**Anti-TB treatment**, ***n*** **(%)**			
Yes	17,493 (99.96)	5,254 (30.03)	12,239 (69.97)
No	7 (0.04)	2 (28.57)	5 (71.43)
**Interval between first symptoms occurrence to visit to a**			
**designated hospital (days)**, ***n*** **(%)**			
0–14	9,569 (54.68)	2,439 (25.49)	7,130 (74.51)
15–29	2,892 (16.53)	824 (28.49)	2,068 (71.51)
30–44	1,776 (10.15)	703 (39.58)	1,073 (60.42)
45-59	622 (3.55)	225 (36.17)	397 (63.83)
≥60	2,191 (12.52)	954 (43.54)	1,237 (56.46)
Unknown	450 (2.57)	111 (24.67)	339 (75.33)
**Interval between visit to a designated hospital and diagnosis**			
**PTB (days)**, ***n*** **(%)**			
0–14	15,047 (85.98)	4,501 (29.91)	10,546 (70.09)
15–29	1,076 (6.15)	284 (26.39)	792 (73.61)
30–44	346 (1.98)	116 (33.53)	230 (66.47)
45–59	163 (0.93)	54 (33.13)	109 (66.87)
≥60	851 (4.86)	295 (34.67)	556 (65.33)
Unknown	17 (0.10)	6 (35.29)	11 (64.71)
**Tnterval between diagnosis of PTB and end of treatment**			
**(days)**, ***n*** **(%)**			
< 180	1,843 (10.53)	552 (29.95)	1,291 (70.05)
180–270	10,832 (61.90)	3,328 (30.72)	7,504 (69.28)
≥270	4,286 (24.49)	1,236 (28.84)	3,050 (71.16)
Unknown	539 (3.08)	140 (25.97)	399 (74.03)
**Migrant population**			
Yes	5,465 (31.23)	1,795 (32.85)	3,670 (67.15)
No	12,035 (68.77)	3,461 (28.76)	8,574 (71.24)
**Treatment outcome**, ***n*** **(%)**			
Treatment completed	11,983 (68.47)	457 (3.81)	11,526 (96.19)
Death	10 (0.06)	2 (20.00)	8 (80.00)
Cure	4,582 (26.18)	4,537 (99.02)	45 (0.98)
Failure	71 (0.41)	30 (42.25)	41 (57.75)
Adverse reaction	19 (0.11)	4 (21.05)	15 (78.95)
Transfer to MDR-TB treatment	82 (0.47)	61 (74.39)	21 (25.61)
Others	753 (4.30)	165 (21.91)	588 (78.09)

### 3.2. Time trend analysis

**Figure 3:**
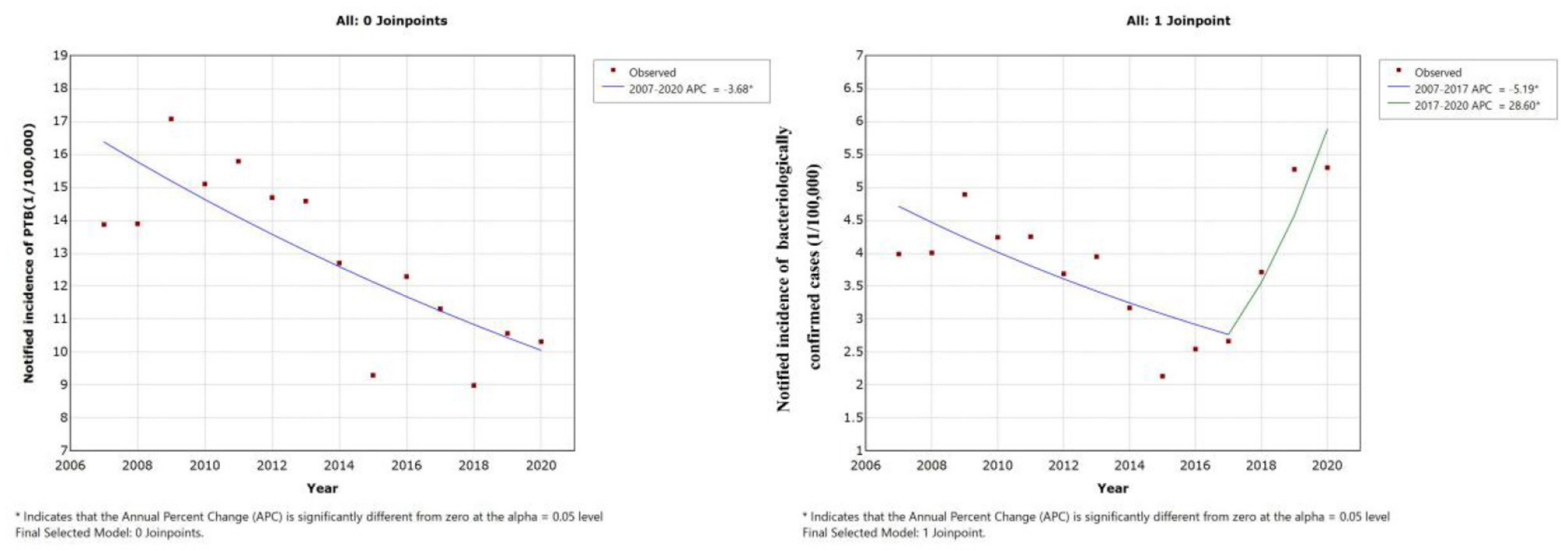
Joinpoint regression analysis of notified PTB incidence in students: **(A)** Time trend of all notified PTB cases. **(B)** Time trend of bacteriologically confirmed PTB cases.

Joinpoint regression analysis revealed a downward trend in notified incidence of PTB cases among students, which decreased from 13.87 cases per 100,000 to 10.32 cases per 100,000 from 2007 to 2020. Furthermore, the annual percentage change was −3.68% (95% CI, −5.4 to −1.9) (Table 2, Figure 3A). However, the notified incidence of bacteriologically confirmed PTB cases showed a different trend and decreased from 3.99 cases per 100,000 to 2.67 cases per 100,000 from 2007 to 2017 but increased from 2.67 cases per 100,000 to 5.30 cases per 100,000 from 2017 to 2020, with APCs of −5.2% (95% CI, −9.1 to −1.1, *P* = 0.018) and 28.6% (95% CI, 2.1 to 62.0, *P* = 0.036), respectively (Table 2, Figure 3B).

**Table 2:** Joinpoint analysis of notified PTB incidence and bacteriologically confirmed PTB incidence in students.

	**Year**	**Annual percent change (95%CI)**	***P***
Notified cases of PTB	2007–2020	−3.68 (−5.40 to −1.90)	0.001
Notified cases of Bacteriologically confirmed	2007–2017	−5.2 (−9.10 to −1.10)	0.018
	2017–2020	28.6 (2.10 to 62.00)	0.036

### 3.3. Spatial autocorrelation analysis

**Figure 4:**
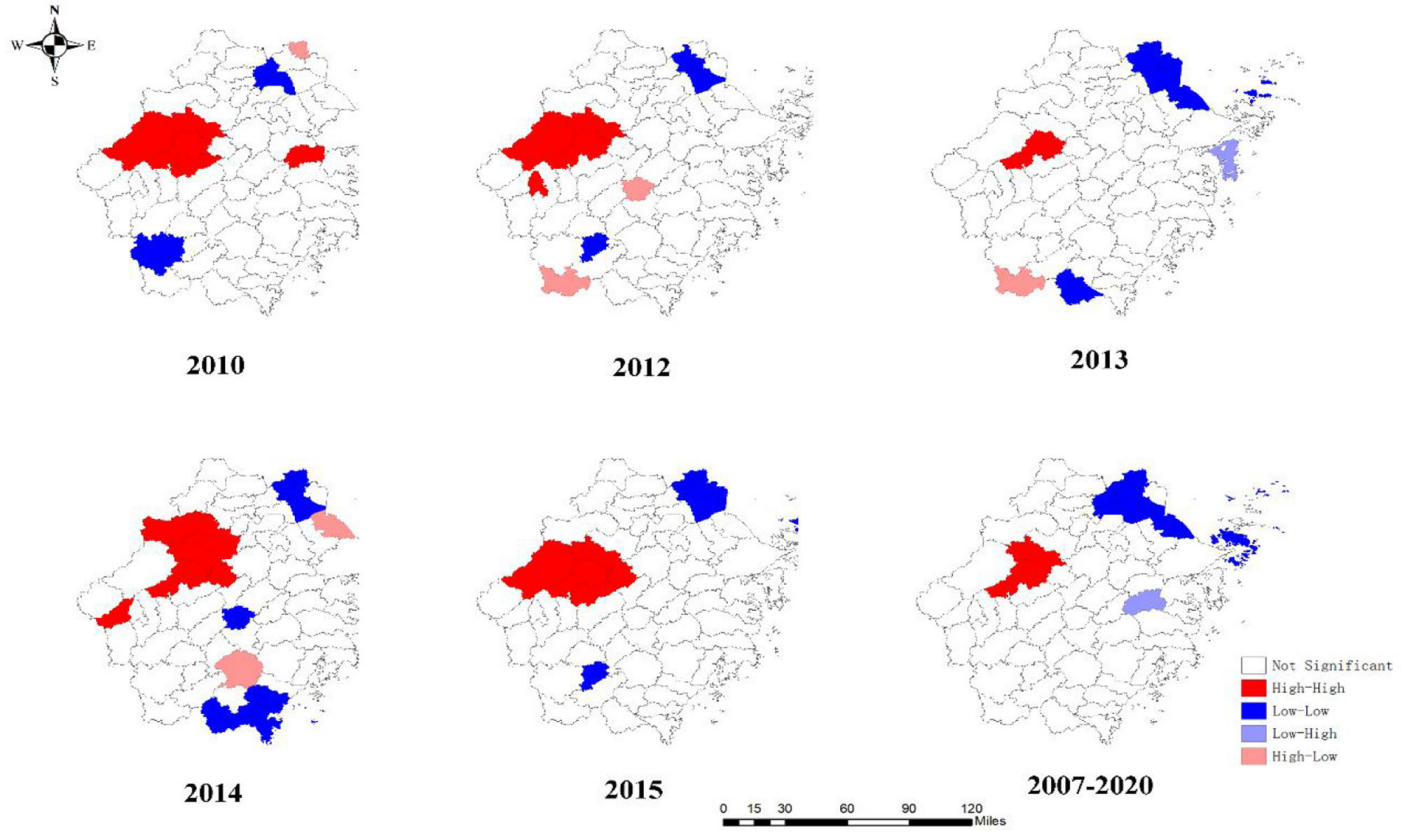
Local spatial autocorrelation of notified PTB incidence among students in Zhejiang Province.

The results of the global autocorrelation analysis indicated that the annual Moran's I was statistically significant in 2007–2010, 2012–2015, 2017, and 2019–2020, while there was no statistically significant difference in 2011, 2016, and 2018 (Table 3). The Moran's I was between 0.180 and 0.407, indicating a spatially positive correlation among students PTB in the Zhejiang Province. The LISA cluster map in county level revealed that hot spot regions of PTB epidemics among students were mainly concentrated at the border of Hangzhou, Jinhua, and Quzhou cities, including Jiande, Tonglu, Chunan, Linan, Lanxi, Pujiang, Changshan, and Kecheng (Figure 4). Although the hot spot regions changed dynamically per year, Jiande and Tonglu remained as hot spots throughout. The cold spots also changed during the study period. For example, the cold spots were mainly concentrated in Jiaxing, Zhoushan, and the western regions of Lishui in 2010, 2012, 2013, and 2015, whereas they were concentrated in Jiaxing, southern Wenzhou, and southeastern Jinhua in 2014.

**Table 3:** Spatial autocorrelation analysis of notified PTB incidence among students in Zhejiang Province.

**Year**	**Moran's *I***	**Z-score**	***P*-value**
2007	0.208	2.649	0.014
2008	0.185	2.367	0.014
2009	0.180	2.289	0.016
2010	0.247	3.131	0.007
2011	0.063	0.950	0.170
2012	0.203	2.531	0.010
2013	0.240	3.230	0.005
2014	0.253	3.032	0.005
2015	0.413	4.998	0.001
2016	0.088	1.652	0.054
2017	0.190	2.459	0.014
2018	0.071	0.950	0.190
2019	0.156	1.929	0.033
2020	0.181	2.187	0.029
2007-2020	0.407	5.007	0.001

### 3.4. Spatial–temporal scan statistic

**Figure 5:**
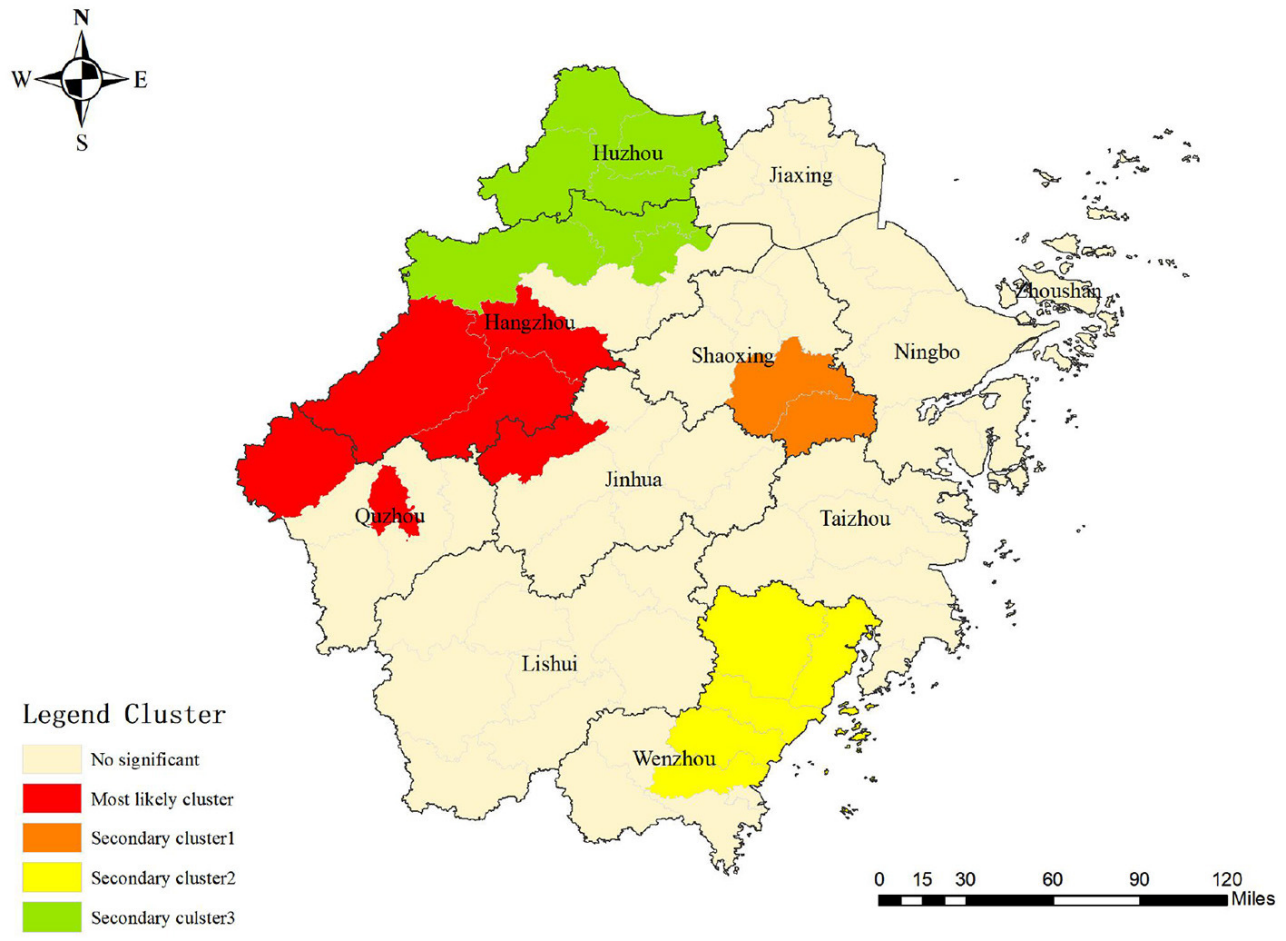
Spatial-temporal clustering result of notified PTB among students in Zhejiang Province.

One most likely cluster and three secondary clusters were identified by a spatial–temporal scan among students in the Zhejiang Province during the study period. For the most likely cluster, the cluster period was from January 2007 to December 2013 and its regions were mainly clustered in the western part of the Zhejiang Province including Jiande, Tonglu, Chunan, Lanxi, Kaihua, and Kecheng, with 691 notified PTB cases. The risk ratio of the most likely cluster was 2.07 (LLR = 14.16, *P* < 0.001) (Table 4 and Figure 5).

**Table 4:** Results of spatial-temporal scanning of PTB among students in Zhejiang, 2007–2020.

**Cluster type**	**Cluster period**	**Coordinates/ radius**	**Number of counties**	**Number of cases**	**Expected cases**	**LLR**	**RR**	***P***
Most likely cluster	2007/1/1–2013/12/31	(29.610699 N,118.883510 E) / 72.02 km	6	691	340.48	142.16	2.07	< 0.001
Secondary cluster1	2007/5/1–2014/4/30	(29.411174 N, 120.964538 E) / 28.42 km	2	391	149.46	136.17	2.65	< 0.001
Secondary cluster2	2007/2/1–2012/5/31	(27.974018 N, 120.670732 E) / 47.25 km	5	1,274	800.82	125.08	1.64	< 0.001
Secondary cluster3	2007/4/1–2013/5/31	(30.622080 N, 119.572321 E) / 70.07 km	7	1,872	1,468.08	56.21	1.31	< 0.001

## 4. Discussion

Spatial–temporal analysis is commonly used to explore disease clustering and to identify high-risk areas of communicable diseases (12). This has significant advantages over conventional epidemiological methods, such as rapid control of epidemics, promotion of health resource allocation, and implementation of health policy (32). Our study used spatial-temporal analysis to reveal the distributions of PTB prevalence among students in the Zhejiang Province, China. Understanding characteristics of the PTB epidemic further among this high-risk group may promote the implementation of efficient interventions to realize the End TB goal in eastern China.

During the study period, the PTB notification rate among students decreased from 13.87 cases per 100,000 to 10.32 cases per 100,000 persons. The notification rate of PTB in the general population in Zhejiang Province decreased by approximately 30% from 2009–2018 (12). Changing trends of PTB epidemics among students and the general population were parallel. These reductions can be attributed to the improvement of pathogenic diagnosis, through prompting the use of molecular diagnostic techniques (such as GeneXpert) and the implementation of new healthcare policies to enhance patient adherence. Meanwhile, in the student group, mass admission screening for PTB may effectively reduce the number of PTB patients when entering schools and reduce the risk of transmission subsequently, potentially playing a vital role in the early identification of active PTB cases (33).

From the age distribution, our results show that PTB cases were mainly concentrated in students aged 13 years and older. Based on the grade feature, it also implied a higher risk of PTB in senior high school and above (14,621) than in junior high school (2,251) students. It is possible that the protective effect of the Bacillus Calmette–Guérin vaccine diminishes over time and a significant protective effect is observed in children aged < 5 years (34). Thus, students may have been confronted with an increased risk of PTB infection and morbidity ~10–20 years after immunization (35). Moreover, increased exposure to PTB and academic pressure may also facilitate progression to PTB. Environmental factors, including crowded and limited classrooms and dormitories ventilation, may also have played important roles in the development of PTB in this population (36, 37). Therefore, strengthening daily surveillance, implementing morning check-ups, and tracking absence caused by illness still should be considered in students.

Our findings demonstrated that delays in health-seeking among students were common (45.32%) from 2007 to 2020 and were lower than national levels among students (47.38%) and older adults (55.1%) (38, 39). However, since long delays lead to the increased transmission of PTB, health education for PTB still should be strengthened in this group.

We observed a downward trend in the notified PTB incidence among bacteriologically confirmed student cases from 2007 to 2017 and a sharp increase from 2017 to 2020. This contrasted with a decreasing trend of total notified PTB cases during the study period. These results were in line with the findings of previous work (40). The decline of notified PTB incidence among students is likely due to the expanded DOTS strategy initiated since 2000 (41). Furthermore, the increased incidence of PTB among bacteriologically confirmed cases since 2017 may be inseparable with the MOH-Gates Foundation TB Control Project in China, which accelerated the application of molecular diagnostic technology for TB finding in Zhejiang Province. The new technology such as GeneXpert assay showed higher sensitivity than classical sputum smear microscopy that enhanced the proportion of PTB cases with positive pathogens (42).

The principal sources of PTB case detection among students were passive findings and referrals during the study period, whereas only a small proportion of PTB cases were detected by active screening. This demonstrated that increased regular screening and health surveillance among students, particularly those with latent PTB infection that refused preventive treatment, should be considered to avoid PTB onset and possibly subsequent outbreak in this special group (43, 44).

Spatial analysis results implied a spatial heterogeneity among students at the county level. The hotpots were mainly distributed in the western cities of the Zhejiang Province, which is consistent with the PTB epidemiological feature among the general population (45). This phenomenon might be attributed to PTB transmission that involved the complex interactions among different population. Besides, these regions have a comparatively high agricultural population with relatively low economic status, along with limited PTB-related knowledge, which were the commonly risk factors for the PTB development (46). Therefore, controlling the PTB epidemics should be given an overall consideration with a priority to lower the disease burden in the entire population. Other special interventions and implementations, including more frequent active screening for students should be considered in these regions in tandem.

In the spatial–temporal analysis, the most likely cluster was mainly concentrated in the western area of the Zhejiang Province which was also similar to the results of the spatial analysis. The clustering period was concentrated only in the first 7 years. After 2014, no cluster was identified, which was attributed to efforts toward PTB prevention and intervention in the Zhejiang Province (40). For instance, comprehensive interventions entailing health policy making for PTB treatment and promotion the application of innovative technology enhanced PTB diagnosis and increased patients' adherence to the treatment, avoiding the potential clusters in the students eventually. In addition, the clustered area shifted from the western regions to the south and north of the Zhejiang Province, implying that the existing of possible unknown risk factors for PTB occurrence. Thus, further in-depth field studies should be conducted to explore the underlying cause.

Our study also has some limitations. First, our data were obtained from the TBIMS and some asymptomatic PTB cases in students might be not seeking for the medical services, which led to an underestimation of PTB incidence among this group. Second, the difference in notification quality in different areas may lead to an inevitable bias, although uniform training was required. Third, in view of the change of administrative regions in counties or districts, we integrated these regions as an entirety, thus influencing further analysis to identify the internal differences.

## 5. Conclusion

In this study, the notified incidence of PTB cases among students showed a downward trend while that of the bacteriologically confirmed cases presented an increase trend since 2017. The risk of PTB among senior high school and above was higher than that of junior high school. Besides, the western area of Zhejiang Province was the high-risk area of student PTB and more comprehensive interventions should be strengthened such as admission screening and routine health monitoring to improve PTB early identification.

## Data availability statement

The datasets presented in this article are not readily available because our data comes from the Tuberculosis Information Management System, and all data is confidential and not available to the public as required. Requests to access the datasets should be directed to KL, kliu@cdc.zj.cn.

## Ethics statement

Our study was approved by the Ethics Committee of the Zhejiang Provincial Center for Disease Control and Prevention (ZJCDC). All records derived from the surveillance system with no private details exempted informed consent by ZJCDC's ethics institutional review board.

## Author contributions

MZ: writing–original draft, methodology, and visualization. SC: data curation and writing–review and editing. DL: visualization, software, and conceptualization. BC: supervision and methodology. YZ: visualization and software. WW: validation, formal analysis, and supervision. QW: data curation, data collection, and validation. KL: supervision, writing–review and editing, and software. JJ: funding acquisition and conceptualization. HW: conceptualization and supervision. All authors contributed to the article and approved the submitted version.
